# Complete Genome Sequence of the Soil-Isolated *Psychrobacillus* sp. Strain AK 1817, Capable of Biotransforming the Ergostane Triterpenoid Antcin K

**DOI:** 10.1128/MRA.01242-20

**Published:** 2021-10-07

**Authors:** Luis B. Gómez-Luciano, Yu-Wei Wu, Chien-Min Chiang, Te-Sheng Chang, Jiumn-Yih Wu, Tzi-Yuan Wang

**Affiliations:** a Office of the VP for Research and Graduate Studies, Universidad Católica del Cibao, La Vega, Dominican Republic; b Graduate Institute of Biomedical Informatics, College of Medical Science and Technology, Taipei Medical University, Taipei, Taiwan; c Department of Biotechnology, Chia Nan University of Pharmacy and Science, Jen-Te District, Tainan, Taiwan; d Department of Biological Sciences and Technology, National University of Tainan, Tainan, Taiwan; e Department of Food Science, National Quemoy University, Jin-Ning Township, Kinmen County, Taiwan; f Biodiversity Research Center, Academia Sinica, Taipei, Taiwan; University of Arizona

## Abstract

The soil bacterium *Psychrobacillus* sp. strain AK 1817 was isolated from a tropical soil sample collected in Taiwan. Strain AK 1817 biotransforms the ergostane triterpenoid antcin K from the fungus Antrodia cinnamomea. The genome was sequenced using the PacBio RS II platform and consists of one chromosome of 4,096,020 bp, comprising 3,907 protein-coding genes, 75 tRNAs, 30 rRNAs, 5 noncoding RNAs (ncRNAs), and 100 pseudogenes.

## ANNOUNCEMENT

*Psychrobacillus* sp. strain AK 1817 is a Gram-positive, motile, endospore-forming, rod-shaped bacterium; of 4,311 soil bacteria isolated using the plating method, only *Psychrobacillus* sp. strain AK1817 was able to biotransform the triterpenoid antcin K into antcamphin E and antcamphin F (1). It was classified into a genus of bacteria in the phylum *Firmicutes*, from the family *Bacillaceae* (1); however, its function/character in the ecosystem remain unknown. This organism’s triterpenoid biotransformation makes it interesting for potential industrial applications.

AK 1817 was isolated from a soil sample collected in southern Taiwan (global position system coordinates 1.345771°N, 103.6801°E) using the Spin Air sampler (IUL, Spain). The colonies were isolated by culturing them on LB agar at 25°C and in LB + 0.2% glucose broth overnight at 25°C prior to DNA extraction. Genomic DNA was purified using the ZR soil microbe DNA kit (Zymo Research, USA) according to the manufacturer’s protocol. After a quality check using the Qubit double-stranded DNA (dsDNA) high-sensitivity (HS) assay kit (Thermo Fisher Scientific, USA) and size selection using the BluePippin system (Sage Science, USA), a library was prepared using the SMRTbell template prep kit v1.0 (Pacific Biosciences), followed by single-molecule real-time (SMRT) sequencing on the PacBio RS II platform.

**FIG 1:**
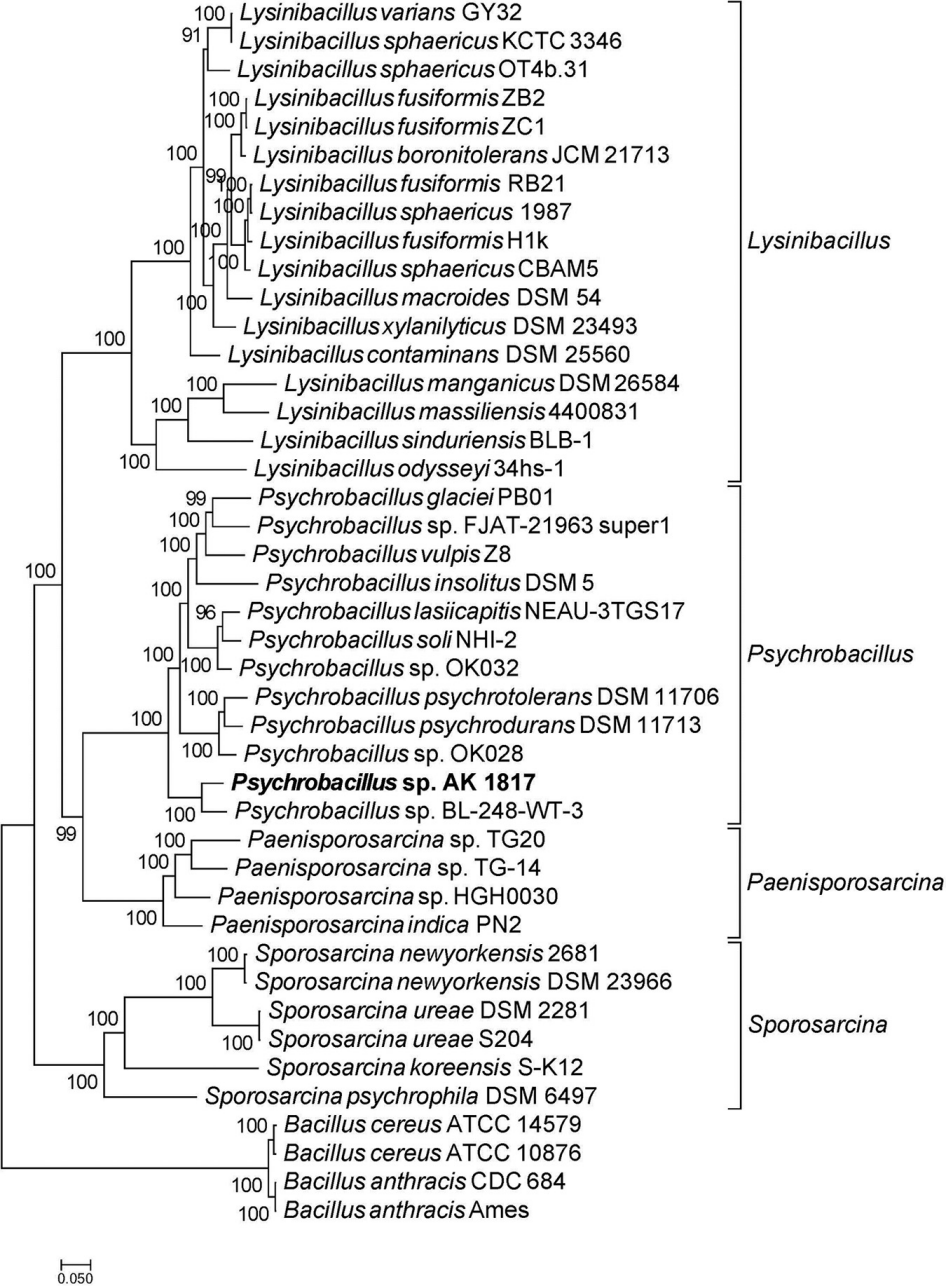
Phylogenomics of *Psychrobacillus* sp. strain AK 1817. This species tree was used to define the gene orthology.

Sequencing generated 149,490 subreads with a total length of 1,232,710,752 bp for a genome coverage of ∼300×. In total, 149,490 subreads were obtained, for which the average, *N*_50_, and maximum read lengths were 8,246, 11,029, and 50,776 bp, respectively. The reads were assembled *de novo* using Canu v1.6 (2) with default settings. Briefly, Canu is capable of correcting read errors and controlling the read quality before assembling the subreads into contigs. The genome contains a single chromosome, which was assembled into one contig (reported by Canu as circular) of 4,096,020 bp, with a GC content of only 36.67%. The genome was annotated using the NCBI Prokaryotic Genome Annotation Pipeline (PGAP) v4.6 (3). It predicted 4,117 genes, of which 3,907 were coding genes, 110 were RNA genes (30 rRNAs, 75 tRNAs, and 5 noncoding RNAs [ncRNAs]), and 100 were pseudogenes. A phylogenetic tree was built using ezTree v0.1 (4), which extracted single-copy marker genes from selected genomes and concatenated the marker genes to build a phylogenetic tree, with four *Bacillus* species as the outgroups using default settings. From the phylogenetic tree (Fig. 1), one can see that AK 1817 was closest to *Psychrobacillus* sp. strain BL-248-WT-3 (GenBank accession number NZ_JABAFC000000000), which was identified from domestic pig feces.

### Data availability.

The complete genome sequence of *Psychrobacillus* sp. strain AK 1817 is available in DDBJ/EMBL/GenBank under accession number CP031739 (BioSample accession number SAMN09845876 and BioProject accession number PRJNA486432). The filtered subreads have been submitted under SRA accession number SRX4910659.

## References

[1] Chiang C-M, Wang T-Y, Ke A-N, Chang T-S, Wu J-Y. 2017. Biotransformation of ergostane triterpenoid antcin K from Antrodia cinnamomea by soil-isolated Psychrobacillus sp. AK 1817. Catalysts 7:299. doi:10.3390/catal7100299.

[2] Koren S, Walenz BP, Berlin K, Miller JR, Bergman NH, Phillippy AM. 2017. Canu: scalable and accurate long-read assembly via adaptive k-mer weighting and repeat separation. Genome Res 27:722–736. doi:10.1101/gr.215087.116. 28298431 PMC5411767

[3] Tatusova T, DiCuccio M, Badretdin A, Chetvernin V, Nawrocki EP, Zaslavsky L, Lomsadze A, Pruitt KD, Borodovsky M, Ostell J. 2016. NCBI Prokaryotic Genome Annotation Pipeline. Nucleic Acids Res 44:6614–6624. doi:10.1093/nar/gkw569. 27342282 PMC5001611

[4] Wu Y-W. 2018. ezTree: an automated pipeline for identifying phylogenetic marker genes and inferring evolutionary relationships among uncultivated prokaryotic draft genomes. BMC Genomics 19:921. doi:10.1186/s12864-017-4327-9. 29363425 PMC5780852

